# A Future English Prospect

**Published:** 1920-01-31

**Authors:** 


### A FUTURE ENGLISH PROSPECT.

A RATHER terrifying prospect of England as a huge garden suburb, with a vanished countryside, was considered by Lord Crewe in an address the other day. He asked whether the structure of society was so altering that, through new conditions of landownership and the imposition of a purely utilitarian colour on rural life, the wilder amenities of the country would entirely disappear in company with many of its surroundings, with the result of turning England into a gigantic garden suburb intersected by a network of admirable roads, and furnished, for hygienic reasons, with a due number of artificial wildernesses, or nature reserves.

Very few Englishmen of to-day would care to live in a spiritless England of that sort, but the tendency undoubtedly will be, as agriculture on a small scale becomes the business of more and more citizens, to abolish the particular picturesqueness afforded by ragged hedges clustered with wild roses, and by undrained bottoms where marsh plants flourish. On the other hand, he believes there is a real desire among the people generally to preserve the special beauties of our English landscape. Happily, in this country the processes of social change are usually slow, so that our children will still be able to wander along winding lanes.

